# Comparison of a rapid immunochromatographic test with a chemiluminescence immunoassay for detection of anti-SARS-CoV-2 IgM and IgG

**DOI:** 10.11613/BM.2020.030901

**Published:** 2020-10-15

**Authors:** Caterina Maria Gambino, Bruna Lo Sasso, Claudia Colomba, Rosaria Vincenza Giglio, Luisa Agnello, Giulia Bivona, Marcello Ciaccio

**Affiliations:** 1 Department of Biomedicine, Neurosciences and Advanced Diagnostics, Institute of Clinical Biochemistry, Clinical Molecular Medicine and Laboratory Medicine, University of Palermo, Palermo, Italy; 2 Department of Laboratory Medicine, University Hospital “P. Giaccone”, Palermo, Italy; 3 Department of Promoting Health, Maternal-Infant. Excellence and Internal and Specialized Medicine (ProMISE) G. D’Alessandro, University of Palermo, Palermo, Italy

**Keywords:** COVID-19, serological test, antibodies, CLIA, immunochromatography

## Abstract

**Introduction:**

The 2019 Coronavirus disease (COVID-19) has been characterized as a pandemic, representing a serious global public health emergency. Serological tests have been proposed as reliable tools for detecting Coronavirus SARS-CoV-2 antibodies in infected patients, especially for surveillance or epidemiological purposes. The aim of this study is to evaluate the agreement between the IgM/IgG rapid assays, based on lateral flow immunochromatographic assay, and the fully automated 2019-nCoV IgM and IgG, based on chemiluminescence immunoassay.

**Materials and methods:**

SARS-CoV-2 antibodies were measured with the BIOSYNEX COVID-19 BSS IgM/IgG test (BIOSYNEX, Illkirch-Graffenstaden, France) and the MAGLUMI CLIA (IgM and IgG) (SNIBE – Shenzhen New Industries Biomedical Engineering, Shenzhen, China) in 70 serum samples from patients with PCR-confirmed diagnosis. The strength of the agreement of the two methods was calculated by using the Cohen Kappa index.

**Results:**

The results showed a good grade of concordance between the two immunoassays with a Cohen’s kappa coefficient of 0.71 (95%CI: 0.54-0.87) for IgG SARS-CoV-2 antibodies and 0.70 (95%CI: 0.53-0.87) for IgM SARS-CoV-2 antibodies. In addition, the rapid assays BIOSYNEX COVID-19 BSS for detecting SARS-CoV-2 antibodies showed a positive likelihood ratio (LR) of 10.63 (95%CI: 2.79-40.57) for IgG and a LR of 6.79 (95%CI: 2.93-15.69) for IgM.

**Conclusion:**

Our results suggest that the immunochromatographic rapid IgM/IgG test and the chemiluminescence IgM and IgG immunoassay have a good degree of concordance, suggesting that both could be considered as useful tools for epidemiologic surveillance.

## Introduction

The 2019 Coronavirus (2019-nCoV or SARS-CoV-2) disease (COVID-19) has rapidly spread across China and to the other countries of the world (*1*, *2*). Since March 2020, COVID-19 has been characterized as a pandemic, representing a serious global public health emergency.

Among infected patients who may display different clinical symptoms, ranging from mild to severe conditions, a discrete amount of individuals remains asymptomatic, being a hidden vehicle, which spreads infection. Accordingly, the World Health Organization (WHO) has stressed the need for research on *in vitro* rapid and effective screening test, in order to quickly identify SARS-CoV-2 infected individuals and prevent contagion (*3*).

To date, the diagnosis of COVID-19 must be confirmed by a molecular test using real-time reverse transcriptase-polymerase chain (RT-PCR) assay on the oropharyngeal or nasopharyngeal swabs for detecting 2019-nCoV genome (*4*). Although RT-PCR represents the gold standard for COVID-19 diagnosis, serological tests are emerging as valid tools for identifying infected subjects, especially for surveillance or epidemiological purposes (*5*). Although there are many commercially available COVID-19 antibody tests, data concerning the performance of these assays are currently limited, and many of these tests have been approved for research use only.

The aim of this study is to compare the BIOSYNEX COVID-19 BSS IgM/IgG rapid test (BIOSYNEX, Illkirch-Graffenstaden, France) with the fully automated MAGLUMI 2019-nCoV IgM and IgG (SNIBE – Shenzhen New Industries Biomedical Engineering, Shenzhen, China), which we have recently implemented in our laboratory. Given the increased demand for rapid serological tests, its comparison with serologically positive or negative results measured by CLIA may be helpful in evaluating the degree of agreement among available immunoassays.

## Materials and methods

### Subjects

A total of 70 patients with PCR-confirmed COVID-19 diagnosis (41 males and 29 females, median age 66 (59-74) years), from April to May 2020, were enrolled in this study. For each patient, the serum sample was obtained during the hospital stay, 7-10 days after nasopharyngeal and oropharyngeal swabs collection. The SARS-CoV-2 IgM and IgG measurement was performed at the Department of Laboratory Medicine, University Hospital “P. Giaccone”, Palermo, Italy. All samples were analysed anonymously. Demographical and clinical data were recorded at admission. All the clinical and biological assessments were carried out in accordance with the Declaration of Helsinki, and the study was approved by the local Ethics Committee. All participants gave written consent.

### Methods

#### CLIA assay

The MAGLUMI 2019-nCoV IgG/IgM assay is a capture CLIA for the quantitative measurement of IgM and IgG antibodies against SARS-CoV-2 in human serum by using the fully automated MAGLUMI analyser (SNIBE – Shenzhen New Industries Biomedical Engineering, Shenzhen, China). According to the manufacturer’s instructions, serum antibodies directed against both virus spike (CoV-S) and nucleocapsid (CoV-N) are detected by using magnetic microbeads coated with ABEI-labelled 2019-nCoV recombinant antigen. As declared by the manufacturer, a threshold of positivity of 1.00 AU/ml was established for both IgM and IgG, whilst the overall indicated reproducibility of assays ranges from 6.8% to 8.7%. Borderline data (≥ 0.95 to < 1.0) were considered reactive.

#### Immunochromatographic assay

The BIOSYNEX COVID-19 BSS assay (BIOSYNEX, Illkirch-Graffenstaden, France) is a rapid, portable qualitative chromatographic assay. The test cassette consists of SARS-CoV-2 recombinant antigens (Spike Protein, as declared by manufacturer) conjugated with colloidal gold; a nitrocellulose membrane strip containing an IgG line (G Line) coated with anti-human IgG, an IgM line (M Line) coated with anti-human IgM, and the control line (C Line) coated with goat-anti-rabbit IgG. It was performed on serum samples according to the manufacturer’s instructions. If a line was observed for IgM and/or IgG, the test was considered positive. If the control line did not appear, the test was invalidated and repeated. Weak signal for IgM and IgG, together or separate, was considered positive.

Ten serum samples were randomly selected and tested six times in order to evaluate the assay repeatability. Two independent operators observed repeatability of 0.83. Both tests are CE (European Community) approved.

### Statistical analysis

Statistical analysis was performed using MedCalc Statistical Software version 15.0 (MedCalc Software Ltd, Ostend, Belgium; https://www.medcalc.org; 2015). Sensitivity, specificity, positive predictive value (PPV) and negative predictive value (NPV) were calculated. Results were considered significant for a P < 0.05. The Cohen Kappa index was calculated to determine the strength of the agreement of the two methods used. Results were interpreted according to the following kappa values: i) 0.01-0.20, slight agreement; ii) 0.21-0.40, fair agreement; iii) 0.41-0.60, moderate agreement; iv) 0.61-0.80, substantial agreement; and v) 0.81-1.00, perfect agreement (*6*).

## Results

The results of the comparison between rapid BIOSYNEX COVID-19 BSS with the MAGLUMI 2019-nCoV for SARS-CoV-2 IgM and IgG detection are reported in Table 1.

**Table 1:** Comparison of rapid BYOSINEX COVID-19 BSS with the MAGLUMI 2019-nCoV for SARS-CoV-2 IgM and IgG detection

		**MAGLUMI 2019-nCov**		
**SARS-CoV-2 IgM**		Positive	Negative	Total
	Positive	24 (34%)	5 (7%)	29 (41%)
**BIOSYNEX COVID-19 BSS**	Negative	5 (7%)	36 (51%)	41 (59%)
	Total	29 (41%)	41 (59%)	70 (100%)
		**MAGLUMI 2019-nCov**		
**SARS-CoV-2 IgG**		Positive	Negative	Total
	Positive	36 (51%)	2 (3%)	38 (54%)
**BIOSYNEX COVID-19 BSS**	Negative	8 (11%)	24 (34%)	32 (46%)
	Total	44 (63%)	26 (37%)	70 (100%)

The comparison between MAGLUMI 2019-nCoV positive/negative *versus* BIOSYNEX COVID-19 BSS positive/negative results yielded an overall concordance of 86% for both IgM and IgG detection, with a Cohen’s kappa coefficient of 0.71 (95%CI: 0.54-0.87) for IgG and 0.71 (95%CI: 0.54-0.87) for IgM.

Table 1 shows the comparison between the rapid test cassette BIOSYNEX and the CLIA MAGLUMI IgG/IgM tests. Overall, the rapid test BIOSYNEX COVID-19 assay showed good accuracy for SARS-CoV-2 IgM and IgG detection compared to the MAGLUMI 2019-nCoV CLIAs (Table 2).

**Table 2:** Accuracy indices for the rapid test BIOSYNEX COVID-19 assay for SARS-CoV-2 IgM and IgG detection compared to the MAGLUMI 2019-nCoV CLIA

**Biosynex Covid-19 BSS** ***vs*** **MAGLUMI 2019-nCov**	**SARS-CoV-2 IgM**	**SARS-CoV-2 IgG**
Sensitivity (95%CI), %	83 (0.64-0.94)	82 (0.67-0.92)
Specificity (95%CI), %	88 (0.74-0.96)	93 (0.75-0.99)
PPV (95%CI) %	83 (0.64-0.94)	95 (0.82-0.99)
NPV (95%CI), %	88 (0.74-0.96)	75 (0.57-0.88)
LR+ (95%CI)	6.79 (2.93-15.69)	10.63 (2.79-40.57)
C-k (95%CI)	0.71 (0.54-0.87)	0.71 (0.54-0.87)
PPV - Positive predictive value. NPV - Negative predictive value. LR - Likelihood ratio. CI - Confidence interval. C-k - Cohen’s kappa.		

## Discussion

In the current study, we compared the results of the rapid test cassette BIOSYNEX COVID-19 BSS for detecting SARS-CoV-2 IgM and IgG with the fully automated MAGLUMI 2019-nCoV CLIA, used as the reference method. Although the MAGLUMI 2019-nCoV assay cannot be considered the serological gold standard test, its analytical performance has been successfully evaluated by Lippi *et al.* and Padoan *et al.* (*7*, *8*). The authors reported a sensitivity of 100% and 88% for detecting SARS-CoV-2 IgG and IgM in COVID-19 patients (*7*, *8*).

In the current study, we showed that the BIOSYNEX COVID-19 BSS IgM/IgG rapid test cassette is congruent with the fully automated MAGLUMI 2019-nCoV, in detecting SARS-CoV-2 antibodies.

Our findings are in accordance with Hoffman *et al.* (*9*). Thus, the BIOSYNEX COVID-19 BSS IgM/IgG rapid test cassette could be used as a quick tool for possible identification of subjects who have had exposure to SARS-CoV-2 infection and thus developed antibodies. Importantly, the target of antibodies detected by BIOSYNEX assay is the viral spike protein binding the spike protein’s receptor-binding domain. As known, any antibody cross-reactivity between common cold human Coronavirus and SARS-CoV-2 would result in false-positive interfering with antibody-based testing and SARS-CoV-2 surveillance. Scientific evidence suggests that the overall specificity of serological assays using only the nucleocapsid protein is poor, whereas assays based on the spike protein are more specific (*10*). Unfortunately, the type of antigen/s is often not reported by *in vitro* diagnostics companies making difficult to understand whether antibodies detected with different assays have a neutralizing effect on the virus. Noteworthy, serum IgM and IgG detection should not be considered as an alternative but as a complementary tool to molecular analysis, providing different clinical information on SARS-CoV-2 infection. While molecular tests allow to directly detecting the virus in the body, serological tests assess the body’s immune response to the infection caused by the virus.

Serological tests are useful potential tools for the rapid screening population, helping to prevent contagion. They are important for activating serological surveillance at the local, regional, and national levels and identifying people who have already had contact with the virus. Compared to molecular assays, these methods save time and are simple to perform, playing an essential role in large-scale testing to evaluate people’s immunity against SARS-CoV2. However, it should be noted that there is no certainty whether people having antibodies are protected from a second infection.

Some limitations shall be disclosed in this study. First, clinical data were not available for all patients due to the limited study period. Second, we used MAGLUMI 2019-nCoV assay as a reference method since no relevant gold standard for serological assays is currently validated for comparative studies. Finally, another limitation is the lack of estimation of the prevalence of the infected population. This is due to the severe Coronavirus situation at the time of the study.

In conclusion, our results suggest that the immunochromatographic rapid BIOSYNEX COVID-19 BSS IgM/IgG test has a good degree of concordance with the MAGLUMI CLIA (IgM and IgG). Thus, it might be considered as an efficient additional tool for characterizing subjects who have had a prior infection and thus developed SARS-CoV-2 antibodies.
